# Supplementary material for: RNAtango: Analysing and comparing RNA 3D structures via torsional angles
Source: PLoS Comput Biol. 2024 Oct 7;20(10):e1012500. doi: 10.1371/journal.pcbi.1012500 (PMC11486365; doi:10.1371/journal.pcbi.1012500)
Supplement: Attachment — Submitted filename: RNAtango-response-to-referees.pdf [file pcbi.1012500.s002.pdf]

We thank the Editor and the Reviewers for all the comments and suggestions. We responded to them to the best of our ability and made the required changes to the manuscript and the RNAtango webserver.

Reviewer #1

In this paper, the author developed a web server for RNA structure analysis according to backbone torsional angles. The server provides three scenarios of analysis, single model, model vs target and model vs model, which are very useful for RNA structure analysis, especially for the assessment of RNA structure prediction. The manuscript was written clearly, but I have a few questions about the work.

1. *In equation 1, the author defined angle P to describe the structure status of the ribose ring. Is there any geometric significance of this angle? What is the meaning of sin 36˚ and sin 72˚? Why not just use v2 to represent the status of ribose ring, I think it's more simple and intuitive.*

   Response: The pseudorotation cycle, established by Altona and Sundaralingam in 1972 (doi:10.1021/ja00778a043), assigns the phase angle P values ranging from 0˚ to 360˚. Using the constants sin 36˚ and sin 72˚, they defined P=0˚ as the C2'-exo-C3'-endo Twist form. Every 18˚ increment in P shifts the ribose ring to a new conformation: P=18˚ corresponds to the C3'-endo Envelope form, P=36˚ to the C3'-endo-C4'-exo Twist form, P=54˚ to the C4'-exo Envelope form, and so forth. Conformations differing by 180˚ in P have opposite signs for all their torsion angles, such as P=90˚ (O4'-endo) and P=270˚ (O4'-exo). We have added a comment to the manuscript to elaborate on interpreting the P angle.

2. *In the last paragraph of page 4, the author mentioned the benefits of comparison of RNA structure in torsion angle space, what about the disadvantages? How reliable it is comparing with RMSD based method?*

   Response: MCQ, relying on averaging, may inadvertently mask specific issues by balancing errors with correctly modeled regions. Currently, MCQ does not differentiate between helical regions and single strands, treating them equivalently. Helical regions, being less flexible in torsion angle distributions and often accurately modeled, can result in lower MCQ values for helix-rich structures, even if the overall fold is incorrect. We recognize this limitation and are actively developing solutions to address it. This complex task involves integrating 2D structural alignment with a weighted MCQ metric to account for models that fold differently from their reference structures. We have added a short paragraph in the manuscript to comment on that topic. Nevertheless, a comprehensive RNA structure quality assessment requires a multi-metric approach, as no single measure provides a complete evaluation. Each metric offers unique insights that, when combined, create a more robust assessment framework. The MCQ metric stands out for its distinct perspective, showing minimal correlation with other measures. In contrast, atomic deviation metrics like lDDT, TM-score, and RMSD are strongly correlated (see Bernard et al., 2024; doi:10.1093/bib/bbae064). For a thorough evaluation of RNA 3D structures against a reference, we strongly recommend using a combination of MCQ, DI (incorporating RMSD and INF), and lDDT (or its correlated alternatives, TM-score or GDT-TS). This multi-faceted approach ensures a comprehensive and reliable assessment of RNA structural quality.

3. *The website is not stable. Sometimes I got the error message "Something went wrong, please try again" after submitting, and sometimes I just can't open the website. After two days of trying, I finally succeeded once, and then the next day it didn't work again.*

   Response: Tool stability is a critical issue, and we appreciate the Reviewer bringing it to our attention. We have enhanced the RNAtango web application's stability through thorough testing and updated restart policies, ensuring quicker recovery from errors or power outages. Additionally, we would like to clarify that during the manuscript review period (specifically over the weekend of August 17-18), there were serviceable power outages at our university that we were not aware of beforehand. Unfortunately, some server machines, including the one hosting RNAtango, failed to automatically restart. As a result, the system was only rebooted by us on Monday, August 19. It is possible that the Reviewer encountered the reported issue while testing the tool during that downtime.

Reviewer #2

The manuscript by Mackowiak et al. describes RNAtango, a web server that offers an analysis of the torsion angles of RNA molecules. The methodology is described in detail and the three examples of intended use are helpful in understanding just exactly what information users of RNAtango can obtain. The manuscript is clearly written, but would benefit from a few clarifications and a more detailed discussion of certain points. Specific comments:

1. *Since (and as mentioned in the manuscript) there are a few computational tools that already determine angular parameters of RNA structure, I would like to see an expanded discussion on how RNA tango compares to those tools, what is the main difference and what additional value it brings. Moreover, other tools in general take as input DNA structures or mixtures of NA molecules as well. Still, I am missing the information if RNAtango is capable of processing other nucleic acids. Can it handle RNA molecules with chemical modifications?*

   Response: RNAtango offers comprehensive support for all nucleic acids, including RNA, DNA, and their hybrids (e.g., PDB ID 1FIX). The software seamlessly integrates modified nucleotides, ensuring accurate representation throughout its interface. In sequence displays, modified nucleotides are presented with their appropriate one-letter codes, as extracted from PDB or PDBx/mmCIF files metadata. The torsion angles' values table and exported CSV files provide full nucleotide names for enhanced clarity. For instance, in the tRNA structure (PDB ID: 1EHZ), nucleotide 10 appears as A.2MG10 in the table, while the sequence panel succinctly displays it as G. We have added this information to the manuscript together with a short discussion on the other available tools for torsion angle-based analyses of RNAs.

2. *As one of the reasons for the use of torsion angle analysis, as specified in the introduction (line 24+), the authors suggest that the RNA folding process depends on the flexibility of the structure determined by angular parameters. I find this description misleading or perhaps just unfortunately phrased since it is the other way around and the torsion angles are restrained and flexibility is limited by energetic conformational minima stabilized with hydrogen bonds and stacking interactions.*

   Response: We agree with the Reviewer's observation regarding the phrasing in this paragraph and have revised it for better clarity.

3. *In a single model mode, is it possible to display regular regions for specific RNA form alongside the analyzed ranges in angular histograms? I believe this would be helpful for the users, to see how much*

certain torsion angles deviate from the typical range. Related to this, please provide such region for A-form RNA in Figure 4.

Response: Figure 4 now incorporates new panels illustrating the structure of the previously examined sequence in its typical A-RNA form. This addition provides a direct comparison between Z- and A-RNA conformations, highlighting their distinct characteristics through 3D visualizations and histograms of key torsion angles. To further enhance user understanding, we have integrated visual markers into the torsion angle histograms on RNAtango's results page, clearly indicating the typical values for A-RNA conformations (see Scheider et al., 2004; doi:10.1093/nar/gkh333). These improvements offer a more comprehensive and intuitive representation of RNA structural differences, enabling users to readily distinguish between Z- and A-RNA forms.

4. *For Figure 5 please add a schematic secondary structure of Zika virus xrRNA next to the heatmap to more easily relate dot-bracket notation of the heatmap with topology. What is the reason for not presenting H1-H7 in numerical order in the heatmap?*

   Response: We have revised our approach to alias assignment, adopting the Reviewer's suggestion to map H1-H7 in sequential order within the Figure 5 legend. This change has been implemented throughout the Figure and accompanying text. Regarding the Zika virus xrRNA's 2D structure schematic, while it is indeed present in Figure 6A, we acknowledge its late introduction may have hindered reader comprehension. To address this, we have modified the text to provide earlier and clearer references to this visual element.

5. *Please avoid using abbreviations (MCQ and LCS-TA) in the abstract.*

   Response: We have removed the abbreviations MCQ and LCS-TA from the abstract**.**

Reviewer #3

RNAtango is a work that enables a complete and comprehensive description of RNA torsional angles from a webserver. With this work, the authors propose the study of RNA structures in three scenarios: single mode, model(s) vs target and model vs model. They strengthen the study with the add of associated metrics for structural quality assessment, the secondary structure as well as clustering with dissimilarity matrix.
The work is validated with examples for each scenarios, showing the promising interests of such tool. The interface is clean and user-friendly, while also being well documented.
Minor concerns:

1. *Fig 7: the size of the numbers might be too high and make the figure less readable.*

   Response: We appreciate the Reviewer's feedback and have decreased the font size of the numerical data to enhance readability and overall clarity of the presentation.

2. *Would it be available and interesting to add for the histogram an option to compare the histogram with the available data from the PDB?*

   Response: RNAtango's result page now features visual markers on its histograms, indicating the torsion angle values characteristic of the A-RNA form—the predominant structure of RNA double helices. These markers serve as valuable reference points for researchers analyzing RNA conformations. While a comprehensive histogram comparing torsion angles across the entire Protein

Data Bank would be ideal, the sheer diversity of nucleotide conformations makes this impractical. Even individual structures like ribosomes exhibit complex distributions of torsion angle values, rendering a meaningful average across all PDB structures nearly impossible to represent clearly. Our approach thus focuses on providing targeted, actionable insights within the context of A-RNA conformations.

3. *Is it possible for users to apply the model vs model with more than 10 models? What if there are 1000 structures to rank?*

   Response: In the Model vs. Model scenario, each loaded structure is treated as a reference structure and a model to compute distances. This is necessary because the MCQ(A, B) calculation is not equivalent to MCQ(B, A). Therefore, if N models are loaded, the system must calculate all possible pairs, resulting in N×N−N comparisons. For example, if 10 structures are loaded, the system computes 90 comparisons, providing results for 90 distinct "reference+model" pairs.
   To ensure an optimal balance between performance and user experience, we have set a limit of 10 models per task. This limit allows us to process each task more quickly, reducing user wait times. Increasing the number of models in a single task could significantly extend processing times, causing delays and inconvenience.
   Our servers have finite computational resources, and effectively managing these resources is crucial. By capping the number of input models at 10, we can prevent system overloads, ensuring the platform remains stable and responsive for all users.
   We recognize that some users may have larger datasets to process. To accommodate this, we encourage submitting multiple tasks sequentially. This approach allows us to maintain system efficiency while enabling users to work with larger datasets in a manageable way.

4. *It could be interesting to add the correlation with RMSD or TM-score (distance-based metrics) with MCQ to highlight the correlation between these distance-based metrics and the torsional-based approach.*

   Response: Correlations between various evaluation measures for RNA, including MCQ, RMSD, and TM-score, have been recently computed and analyzed by Bernard et al., 2024 (doi: 10.10.93/bib/bbae064). The authors of that paper concluded, "The MCQ metric seems to be the only metric that is not correlated to the others. Atomic deviation metrics tend also to be correlated: lDDT, TM-score, RMSD and RMSD. We advise using MCQ as an evaluation metric to assume RNA nativity with DI (RMSD and INF) and lDDT (or TM-score or GDT-TS, as they are correlated). It provides a complementary set of metrics that assess RNA 3D structure evaluation compared to a reference structure.” We have added a short paragraph in the Introduction that references these findings.

5. *Is it a way to download the angles predicted with single mode for a user?*

   Response: Yes, all data generated by RNAtango is downloadable. In the Single model scenario, you can download all torsion angle values by clicking the “Download .csv” button beneath the table containing these angles. The table is collapsed by default. To view it, click on the bar under the “Torsion angle values” label. Once expanded, the table with all the angle values will be visible, along with the download button (see figure below). However, not all users seemed aware they could click on the bar to expand the table. Therefore, we’ve added a “Click to expand” label to the bar to improve this, making it more intuitive.
